# Feasibility of Induction Docetaxel, Cisplatin, 5-Fluorouracil, Cetuximab (TPF-C) Followed by Concurrent Cetuximab Radiotherapy for Locally Advanced Head and Neck Squamous Cell Carcinoma

**DOI:** 10.3389/fonc.2013.00005

**Published:** 2013-01-29

**Authors:** Nikolaos Charalambakis, Vassilis Kouloulias, Helena Vaja, Dimitrios Pectasides, Theodoros Rampias, Theofanis Economopoulos, Panagiotis Katsaounis, Spiros Siolos, Valentina Bartzi, Christos Perisanidis, Konstantinos Laschos, Pavlos Maragoudakis, Konstantinos Proikas, Nikolaos Papadimitriou, Nikolaos Papadogeorgakis, Helen Georgopoulou, Anna Zygogianni, Ioli Artopoulou, Eleni Pappa, George Dimitriadis, Amanda Psyrri

**Affiliations:** ^1^Second Department of Internal Medicine Propaedeutic, Oncology Section, Attikon University Hospital, University of Athens Medical SchoolAthens, Greece; ^2^Department of Radiation Oncology, Attikon University Hospital, University of Athens Medical SchoolAthens, Greece; ^3^Second Department of Internal Medicine, Oncology Section, “Hippokration” Hospital, University of Athens School of MedicineAthens, Greece; ^4^Department of Molecular Biophysics and Biochemistry, Yale UniversityNew Haven, CT, USA; ^5^Department of Cranio-, Maxillofacial, and Oral Surgery, Medical University of ViennaVienna, Austria; ^6^Second Department of Otorhinolaryngology, Medical School, Attikon General University HospitalAthens, Greece; ^7^Department of Oral and Maxillofacial Surgery, Dental School, “Evangelismos” General Hospital, University of AthensAthens, Greece; ^8^Iaso Private Hospital, Radiotherapy DepartmentAthens, Greece; ^9^Department of Prosthodontics, National and Kapodistrian University of Athens, School of DentistryAthens, Greece

**Keywords:** HNSCC, TPF-C, cetuximab radiotherapy, toxicity and outcome, mutation analysis, *PIK3CA*, HPV DNA

## Abstract

**Purpose:** To report our experience with a sequential regimen of induction TPF-C followed by radioimmunotherapy with cetuximab in patients with locally advanced head and neck squamous cell carcinoma (HNSCC).

**Patients and Methods:** Toxicity and outcome was retrospectively analyzed in 22 patients receiving sequential therapy with induction TPF-C followed by radioimmunotherapy between October 2008 and December 2011. Outcome was estimated using Kaplan–Meier analyses. In addition, we performed mutation analysis for *PIK3CA* genes and high risk HPV DNA detection using PCR.

**Results:** Mean time of follow-up was 16 months. Six patients were TNM Stage III, 15 patients IV (IVA or IVB), and one patient Stage II with bulky disease. During TPF-C, Grade 3 and 4 toxicities occurred in eight patients, dose modifications in seven, delays in one, and unplanned admissions in five. Clinical tumor response was documented in 18 of the 21 patients who completed at least three cycles of TPF-C with three patients developing complete response and 15 partial responses. Grade 3/4 mucositis was observed in six patients. At a median follow-up of 19 months, 13 patients were alive and nine had died including seven patients as a result of disease persistence or recurrence and two as a result of unrelated causes. *PIK3CA* mutations were not identified and our two oropharynx cases were HPV negative.

**Conclusion:** The combination of induction TPF-C with concurrent cetuximab radioimmunotherapy in patients with locally advanced HNSCC is tolerable, with encouraging efficacy.

## Introduction

Head and neck squamous cell carcinoma (HNSCC) is the sixth most common cancer worldwide. Approximately 60% of patients present with locally advanced disease (Al-Sarraf, 2002; Seiwert and Cohen, 2005). Tobacco use or/and alcohol consumption are implicated in the majority of cases. High risk Human papilloma virus (HPV) infection, mainly type 16, accounts for a subset of HNSCC, particularly oropharyngeal squamous cell carcinomas (OSCC; Gillison et al., 2000; Weinberger et al., 2006). HPV positivity confers a 60–80% reduction in risk of death from disease compared to similarly treated HPV negative cancers (Gillison, 2010). Median survival time for HPV negative patients was 21 months and not reached for HPV positive patients in TAX324 clinical trial of sequential chemotherapy regimen (Posner et al., 2011). The 5-year progression-free (PFS) and overall survival (OS) rates for HPV negative patients were 28 and 35%, respectively while PFS and OS for HPV positive patients were 78 and 82%, respectively. It seems that even with aggressive sequential therapy programs, HPV negative patients have an extremely poor outcome. An important goal of research strategies for poor prognosis HPV negative cancers will be optimization of treatment regimens with the incorporation of novel active agents.

Multimodality treatment approaches including chemotherapy and radio immunotherapy have been extensively studied (Argiris et al., 2010; Jensen et al., 2011). A meta-analysis including more than 17,000 patients (Pignon et al., 2009) demonstrated a 6.5% absolute survival benefit for concurrent chemoradiotherapy and an 11% survival benefit for concurrent cisplatin chemoradiotherapy. Concurrent high-dose cisplatin plus radiation therapy represents the widely accepted standard of care for patients with stage III through IVB HNSCC (Adelstein et al., 2003; Forastiere et al., 2003). A smaller benefit was seen in meta-analysis with induction chemotherapy. Two phase III randomized studies tested the incorporation of docetaxel to the standard PF induction regimen (Posner et al., 2007; Vermorken et al., 2007). Both studies have demonstrated the superiority of TPF against PF in terms of PFS and OS. TPF is considered the new standard regimen for induction chemotherapy.

Cetuximab, a monoclonal antibody targeting the epidermal growth factor receptor (EGFR), is an active agent in head and neck cancer. Bonner et al. (2006, 2010) showed that cetuximab combined with radiotherapy (RT) significantly improves locoregional recurrence rate, progression-free and OS compared with RT alone in locally advanced HNSCC. Cetuximab plus RT, however, has not been compared to the current standard, cisplatin plus RT, in the setting of a phase III randomized trial.

The EXTREME study in recurrent/metastatic setting showed that cetuximab combined with platinum plus 5-Fluorouracil (PF) improves outcome measures (Vermorken et al., 2008). To increase efficacy, TPF plus cetuximab (TPF-C) appears an appealing induction regimen. The combination of TPF-C induction chemotherapy followed by cetuximab radioimmunotherapy in order to maximize efficacy and reduce toxicity is appealing therapeutic strategy.

A consistent mechanism of resistance to cetuximab has not been described in HNSCC. Breast cancers with either activating mutations in *PIK3CA* or with PTEN loss are resistant to treatment with the Her2/Neu targeting antibody, trastuzumab (Berns et al., 2007). Jhawer et al. (2008) also showed that *PIK3CA* and *Ras/BRAF* mutation status may stratify colon cancer patients that may benefit from cetuximab. Taken together, these data support the notion that the mutation status of the PI3K signaling pathway should be considered before treatment with EGFR-targeted therapy.

Here, we report our experience of treating selected fit patients with locally advanced HNSCC with 3–4 cycles of induction with modified TPF-C regimen followed by concurrent weekly cetuximab (400 mg/m^2^ loading dose before RT and 250 mg/m^2^ weekly during the 7 weeks of RT), demonstrating the feasibility of this approach along with encouraging evidence of efficacy. In addition, we sought to determine the incidence of *PIK3CA* activating mutations in this small cohort of patients with HNSCC.

## Materials and Methods

### Eligibility for TPF-C induction chemotherapy

**Figure 1:**
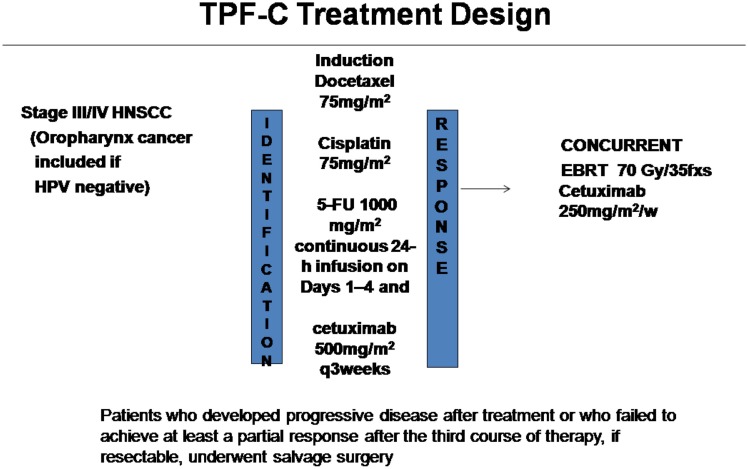
**Treatment Schema**.

In October 2008 the Attikon Hospital approved the use of induction TPF-C chemotherapy followed by concurrent cetuximab-based radioimmunotherapy for the management of patients with locally advanced HNSCC (Figure 1). TPF-C consisted of docetaxel 75 mg/m^2^ on day 1, cisplatin 75 mg/m^2^ on day 1, 5-Fluorouracil dose of 1000 mg/m^2^/day on days 1–4 and cetuximab 500 mg/m^2^ on day 1 of 21 day chemotherapy cycle. Before treatment the records of each patient were reviewed independently to ensure compliance with the following eligibility criteria: (1) biopsy-proven squamous cell carcinoma of the oral cavity, oropharynx (HPV negative), hypopharynx, larynx, or unknown primary; (2) TNM Stage II with bulky disease, III, or IV (IVA or IVB) according to American Joint Committee on Cancer Classification (Sobin and Compton, 2010); (3) Unresected tumor prior to chemoradiotherapy due to technical unresectability and/or low surgical curability and/or organ preservation (4) ECOG performance status (PS) 0–1; (5) adequate bone marrow function; (6) adequate liver function; (7) no prior chemotherapy or RT for HNSCC.

### Data collection

Consecutive patients treated with TPF-C chemotherapy for HNSCC from October 2008 until December 2011 were identified from medical records for analysis. Data were obtained from retrospective review of chemotherapy and RT medical records, paper notes, and RT treatment charts.

### Pretreatment assessment

All patients were treated by the head and neck multidisciplinary team, according to protocol. Within this protocol, all patients were investigated and staged with endoscopy, biopsy, CT scan, and/or MRI of the head and neck region, and CT of the thorax. The option of a prophylactic gastrostomy was routinely discussed.

### Induction chemotherapy

Induction chemotherapy was planned to be three to four cycles (number of cycles at clinician discretion) of docetaxel 75 mg/m^2^ as a 1-h infusion on Day 1, cisplatin 75 mg/m^2^ as a 4-h infusion on Day 1, 5-Fluorouracil 1000 mg/m^2^ as a continuous 24 h infusion on Days 1–4 and cetuximab 500mg/m^2^ on day 1 three-weekly. Carboplatin area under the concentration curve 5 (AUC 5) was substituted for cisplatin in patients older than 65 years of age and in those with creatinine clearance less than 55 mL/min. All patients were given adequate hydration and antiemetics (dexamethasone and 5-hydroxytryptamine-3 antagonists). All patients were given dexamethasone to prevent docetaxel-related hypersensitivity reactions, skin toxic effects and fluid retention and prophylactic antibiotics on day 5 of each cycle for 10 days. Clinical assessment of tumor response was made by a combination of clinical and imaging (CT or MRI) assessment. Imaging assessment was performed after the third cycle of induction chemotherapy. Toxicity was prospectively recorded according the National Cancer Institute version 3.0 grading system for chemotherapy toxicity^1^.

A cycle could be delayed for up to 2 weeks to allow for a reduction in toxicity of grade 3 or more to a severity of grade 1 or less (with the exception of alopecia, fatigue, and nail changes). Delays beyond 2 weeks required discontinuation of TPF-C. Reductions in docetaxel dose were planned for grade 3 and 4 neutropenia, skin toxicity, and impaired liver function tests. Reductions in the cisplatin dose were made for peripheral sensory/motor neurotoxicity, ototoxicity, or renal impairment. Modifications in the 5-fluorouracil dose were made for diarrhea and mucositis.

### Radiotherapy

All patients were treated with three-dimensional conformal RT. Patients were simulated supine with a thermoplastic mask for immobilization. Planning CT images were obtained at 3 mm intervals, and CT data were loaded into the ECLIPSE VARIAN treatment planning system. Virtual simulation was done by using the PROSOMA system. Prechemotherapy imaging was used to define target volumes in line with recent guidance (Forastiere et al., 2003). The clinical target volume included primary site and bilateral level Ib, II, III, IV, and V lymph nodes. Retropharyngeal lymph nodes were variably included, depending on tumor site and stage. Treatment was routinely planned with a two phase conformal technique of two lateral parallel opposed 6-MV photon fields with multiple field-in-fields, with a matched anterior neck field. The posterior border of the lateral photon fields was brought anterior to spinal cord to avoid cord toxicity after 44 Gy in 22 fractions. Matched electron fields were applied to the posterior neck. Standard doses were 70 Gy in 35 fractions over 7 weeks, with 50 Gy in 25 fractions over 5 weeks to the matched anterior neck. The quality assurance procedure included portals once per week and in selected cases *in vivo* dosimetry in the junction between anterior neck and lateral field. RT-related toxicity was prospectively recorded using the Radiation Therapy Oncology Group (RTOG) toxicity criteria (Cox et al., 1995).

### Concurrent radioimmunotherapy

Concurrent radioimmunotherapy consisted of cetuximab 400 mg/m^2^ loading dose before RT and 250 mg/m^2^ weekly during the 7 weeks of RT (70Gy). Toxicity was prospectively recorded according to the National Cancer Institute version 3.0 grading system for chemotherapy toxicity (see text footnote 1).

### Response assessment and follow-up

Patients were initially evaluated at the head and neck tumor board where they were formally staged. Tumor response was assessed after three cycles TPF-C and 3 months after completion of radioimmunotherapy. Evaluation of tumor response was by clinical examination, endoscopy, and CT or MRI imaging of the primary site and the neck; examination under anesthetic and biopsies were performed in the event of any persistent clinical or radiologic abnormality. Response evaluation was based on RECIST criteria. Organ preservation was one goal of the treatment but was secondary to attaining cure. Patients who developed progressive disease after treatment or who failed to achieve at least a partial response after the third course of therapy, if resectable, underwent salvage surgery. Patients underwent repeat examination under anesthesia 8 weeks after CRT, at which time the primary tumor site was examined (and biopsied if abnormal). Observation was recommended for patients with initial N^+^ disease who achieved complete or partial response after chemoradiotherapy. Patients were followed for relapse and survival.

### Second primaries

Second primaries were defined based on the Warren and Gates (1932) criteria. A new cancer of different histology, one of identical histology diagnosed beyond 3 years after treatment of the primary tumor, or one separated from the initial primary tumor by greater than 2 cm of clinically normal epithelium were all considered second primaries.

### Molecular analysis of tumor specimens

#### Genomic DNA extraction

Genomic DNA was extracted from 10 mm paraffin-embedded sections of the tumor samples. Slides were microscopically examined and tumor areas were marked and carefully dissected under microscopic observation. DNA extraction from dissected material was performed using the EX-WAX^TM^ Paraffin-embedded DNA Extraction kit (Millipore, Temecula, CA, USA) according to the manufacturer’s tissue protocol.

#### PIK3CA mutation detection

Sequences of *PIK3CA* (exons 9 and 20) were polymerase chain reaction (PCR) amplified (using primer pairs are listed in Table 2). PCR reactions were performed in 20 μL final volume, containing 50 ng of genomic DNA, 10 μl of Qiagen HotStarTaq Master Mix Kit (Valencia, CA, USA) and 1 μM of forward and reverse primers. After 3 min of initial denaturation, the PCR mixtures were subjected to 35 cycles of denaturation for 30 s at 95°C, annealing for 45 s at variable temperature according to the amplicon (see Table 1) and extension for 45 s at 72°C. A final extension period of 3 min at 72°C was performed to complete the reaction.

**Table 1:** **Primers**.

Target	Sequence (5′-3′)	Annealing temperature (°C)
PIK3CA (exon 9)	fw: GTCTTAGATTGGTTCTTTCCTGTC re: ATGGCAAAGAACACAAAAGG	56
PIK3CA (exon 20)	fw: TGGGGTAAAGGGAATCAAAAG re: CCTATGCAATCGGTCTTTGC	62
High risk HPV (GP5 + /GP6 +)	fw: TTTGTTACTGTGGTAGATACTAC re: CTTATACTAAATGTCAAATAAAAAG	40
HPV 16 E7	fw: CGGAATTCATGCATGGAGATACACCTACAT re: CGGGAAGCTTATGGTTTCTGAGAACAGATGG	58
Human β-globin	fw: GGAGAACTCTGCCGTTACTGC re: TTGGTCTCCTTAAACCTGTCTTGT	56

**Table 2:** **Demographic characteristics**.

**Age**
<65 years	4
≥65 years	18
**Sex**
Male	20
Female	2
**Smoking**
No	2
Yes	20
**Etoh**
No-social	9
Mild-heavy	13
**Hpv status**
(−)	22
(+)	0

Polymerase chain reaction products were then purified using QIAquick PCR purification kit (Qiagen), sequenced on Applied Biosystems 3730 Genetic Analyzer (Foster City, CA, USA) and analyzed by using Sequencher v 4.9 software. Results obtained were confirmed on both sense and antisense strands.

#### Detection of high risk HPV DNA by PCR

Detection of high risk HPV was performed by GP5+/6+ PCR assay on 50 ng DNA as described by de Roda Husman et al. (1995). Detection of HPV 16+ samples was performed by E7-type 16 specific PCR as described by Jiang and Milner (2002).

To check the DNA quality, the specimens were amplified with beta-globin primers. As positive control, serial dilutions of DNA isolated from the cervical carcinoma cell line SiHa (HPV 16 positive) were included. Reactions without template were used as negative controls.

Polymerase chain reaction products were analyzed on a 2% agarose gel stained with ethidium bromide and visualized by UV-transillumination. Oligonucleotide sequences are shown in Table 1.

### Statistical analysis

The response rate was expressed as the proportion of patients who demonstrated a CR and/or PR. OS was measured from the day of histologic diagnosis until death of any cause. Progression-free survival (PFS) was calculated from the date of histologic diagnosis until date of progressive disease or death of any cause. PFS and OS times were summarized using Kaplan–Meier product limit curves using log-rank analysis to determine statistical significance. All calculations and analyses were carried out with SPSS 17.0 for Windows (SPSS Inc., Chicago, IL, USA).

## Results

### Patients and tumor characteristics

Twenty-two patients were treated between October 2008 and December 2011 with induction TPF-C at Attikon Hospital. The demographic characteristics of the patients are shown in Table 2. Mean follow-up was 16 months (range, 6–38 months). Mean age was 55 years (range, 35–77 years). Twenty patients were male; 20 were current or ex-smokers (>10 pack years); 13 had a history of high or moderate alcohol consumption. All patients were ECOG PS 0 or 1. All patients had pathologically proven squamous cell carcinoma. Tumor subsite distribution and pathologic grade are summarized in Table 3. The TNM classification for these patients is summarized in Table 4.

**Table 3:** **Summary of tumor subsite and histologic grade**.

Parameter	*n* (total = 22)
**PRIMARY TUMOR SITE**	
Oral cavity	8
Oropharynx	2
Larynx	7
Hypopharynx	3
Unknown primary	2
**HISTOLOGIC GRADE**	
Well differentiated	1
Moderately differentiated	10
Poorly differentiated	7
Unclassified	4

**Table 4:** **Summary of TNM distribution**.

*N* stage	0	1	2A	2B	2C	3	Total
*T* stage
1	–	1	–	–	–	–	1
2	1	1	–	–	1	–	3
3	3	1	–	4	1	–	9
4	4	–	–	–	2	1	7
*X* (occult primary)		–	–	–	1	1	2
Total	8	3	–	4	5	2	22

### Induction chemotherapy with TPF-C: Delivery, toxicity, and clinical response

The toxicity of TPF-C is summarized in Table 5. All patients were planned to receive 3–4 cycles of induction TPF-C chemotherapy. However, one patient received only one cycle of TPF-C, due to thrombosis of the jugular vein (port-a-cath insertion site) which required prolonged hospitalization and TPF-C discontinuation. One patient received five cycles due to individual physician preference. In three patients, cisplatin was substituted by carboplatin. Two of them were older than 65.

**Table 5:** **Summary of toxicity of TPF-C chemotherapy**.

Toxicity	Grade 1	Grade 2	Grade 3	Grade 4
Neutropenia	3	1	3	1
Nausea/vomiting			1	
Infection			1	
Cardiac toxicity			1	
Diarrhea			1	
DVT			1	
Pulmonary embolism				1
Allergic reactions			1	
Transaminitis	1			
Mucositis	3	1		
Skin toxicity	3	3		
Renal toxicity	1			

Grade 3 and 4 toxicities during induction TPF-C were recorded in 8 patients (Table 4). Delay in administration of TPF-C occurred in one patient due to grade 4 neutropenia. Dose modifications were made in a total of seven patients. Modifications included omission of 5-FU for two cycles in one patient due to cardiac ischemia, omission of day 3 and 4 of 5-FU for one cycle due to grade 3 diarrhea in one patient, omission of docetaxel for one cycle due to grade 3 allergic reaction in one patient and 20% dose reductions in four patients secondary to Grade 3/4 toxicity. In one patient cisplatin was substituted by carboplatin due to decline in renal function.

Five unplanned admissions occurred during induction chemotherapy with TPF-C. Duration of admission varied from 5 days to over a month. Reasons for admission included pulmonary embolism (*n* = 1), febrile neutropenia (*n* = 1), intractable nausea, and vomiting (*n* = 1), febrile thrombophlebitis of jugular vein (*n* = 1) and extensive tongue bleeding due to tumor progression (*n* = 1).

Tumor response was assessed clinically at the end of three cycles of induction chemotherapy. Clinical tumor response (CR or PR) was documented in 18 of the 21 patients who completed at least three cycles of TPF-C with three patients developing complete response and 15 partial responses. One patient had stable disease and two patients developed disease progression. One patient with progressive disease went on to salvage surgery (laryngectomy) followed by RT and the other one developed extensive tongue bleeding and was treated with RT alone.

### Time from completing chemotherapy to commencing RT

Of the 21 patients who completed induction TPF-C, 19 patients who attained at least SD with TPF-C chemotherapy went on to receive radioimmunotherapy with cetuximab. The median time from the administration of the final cycle of TPF-C to delivery of the first fraction of RT was 25 days (range, 13–37 days). Four patients began RT ≤ 21 days, 3 ≤ 28 days, and 12 ≤ 35 days after the final administration of TPF-C.

### RT delivery

Of the 19 patients treated with radical RT schedules, the majority received 70 Gy in 35 fractions prescribed over 7 weeks. All 19 patients completed their prescribed radical RT regimen.

### Administration of radioimmunotherapy

From the original cohort of 22 patients, 19 received radioimmunotherapy with cetuximab. Two patients received four cycles of concurrent cetuximab, four received five, six received six, and seven patients received seven cycles. Four patients experienced delays in delivery of cetuximab.

The toxicity during radioimmunotherapy with cetuximab is summarized in Table 6. Grade 4 complications were recorded in one patient. Non-hematologic grade 3 and 4 toxicities were observed in eight patients. Non-hematologic grade 3 toxicities included nausea and vomiting, fatigue/weight loss, skin toxicity, and mucositis. Hematologic grade 3 or 4 toxicity was not encountered in any patient.

**Table 6:** **Toxicity in administration of concurrent cetuximab radiotherapy**.

Toxicity	Grade 1	Grade 2	Grade 3	Grade 4
Nausea/vomiting		1	1	
Fatigue-weight loss	1	2	1	
Skin toxicity	2	6	1	
Mucositis	1	3	5	1
Anemia	1			

Cetuximab-related toxicity was recorded according to the NCI-CTCAE classification system (version 3). Cetuximab-induced skin toxicity was found in nine patients, eight of whom had grade 1–2 skin toxicity, and 1 grade 3. RT-related toxicity was recorded according to Acute Radiation Morbidity Scoring Criteria of the RTOG. Mucosal toxicity (mucositis) was the most common and was observed in all the patients. Grade 4 mucositis was recorded in one patient, grade 3 in five patients, and grade 2 in five patients. One unscheduled admission for one day during concurrent radioimmunotherapy occurred due to grade 4 mucositis.

### Assessment of response 3 months after completion of RT

Of the 19 patients who completed radical RT plus cetuximab, four achieved complete response to treatment. Fourteen patients had partial response and one patient had stable disease.

### Mutation analysis of tumor samples

Fifteen of 22 patients had formalin fixed paraffin-embedded tissue (FFPE) available for molecular analysis. Thirteen of 15 FFPE samples had amplifiable DNA. Mutations of *PIK3CA* gene within the helical and kinase domains (exons 9 and 20) can cause constitutive activation of PI3K and the consequent activation of AKT pathway (Du et al., 2012). We therefore analyzed all cases for somatic mutations within the two hotspot regions (exons 9 and 20) of *PIK3CA* gene, where >80% of the reported mutations are found (Qiu et al., 2006). However, none of cases were found to harbor PIK3CA activating somatic mutations (data not shown). Our retrospective analysis included only two oropharynx cases which were both HPV negative.

### Outcomes

**Figure 2:**
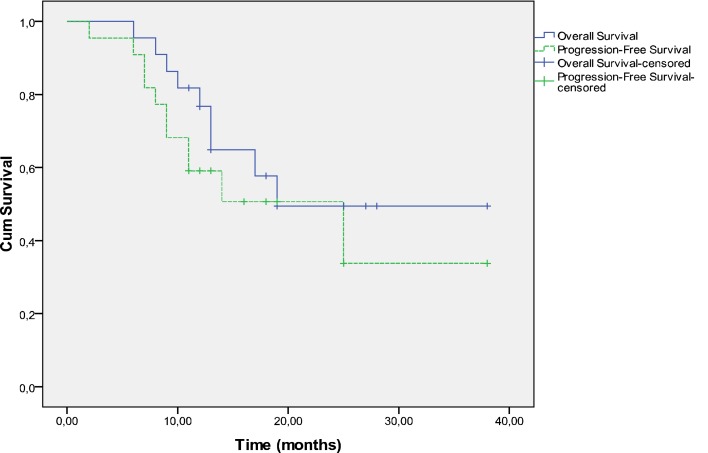
**Progression-free and overall survival**.

Survival and PFS are shown in Figure 2. The 1-year OS rates are 76.7% (95% CI, 60.7–96.9%) and the 1-year PFS was 59.1% (95% CI, 41.7–83.7%), respectively.

At a median follow-up of 19 months, 13 patients were alive and nine had died including seven patients as a result of disease persistence or recurrence and two patients as a result of unrelated causes; one died of pneumonia 9 months after diagnosis and a second in less than a month after treatment completion due to starvation as a consequence of psychotic depression. The median time for progression-free survival was 25 months. Nine patients have had documented disease progression. The first site of progression was locoregional in six patients. Three patients experienced treatment failure distantly without documented locoregional failure. Of the nine patients who relapsed, seven had a relapse in ≤12 months and two in >12 months. One patient with local recurrence underwent laryngectomy followed by RT and is still free of disease. One patient with hypopharynx cancer developed tonsillar cancer as a second primary which was managed with surgery.

## Discussion

The data presented here demonstrate the feasibility of induction TPF-C followed by cetuximab-radioimmunotherapy in patients with locally advanced HNSCC. We report that induction with a modified TPF-C regimen followed by radioimmunotherapy with cetuximab is a safe and active sequential therapy regimen.

Four studies have evaluated induction TPF-C in HNSCC. The feasibility of adding cetuximab to induction TPF was first demonstrated in a phase I study conducted at Dana Farber Cancer Institute (Haddad et al., 2009). The primary objective of the study was to determine the maximum tolerated dose (MTD) of 5-Fluorouracil in TPF regimen when combined with standard dose cetuximab for induction treatment of locally advanced HNSCC. Of the three 5-FU dose levels studied (750, 850, and 1000 mg/m^2^) 850mg/m^2^ daily as a continuous infusion days 1–4 of each chemotherapy cycle was identified as the MTD. At this dose level three of 13 patients developed a dose-limiting toxicity (mucositis, diarrhea, febrile neutropenia). An average of seven cetuximab doses was administered of the nine intended. All evaluable patients attained a partial radiographic response after induction and sixteen of twenty (80%) patients who underwent biopsy of the primary site before the initiation of CRT developed a pathological CR. All 28 patients went on to receive concurrent chemo RT per treating institution practice.

A similar toxicity pattern was seen in an EORTC Phase II study, which combined cetuximab to the European TPF regimen (cisplatin 75 mg/m^2^ and docetaxel 75 mg/m^2^ on day 1 followed by 5-FU 750 mg/m^2^/day as a continuous infusion for 5 days). After four cycles of TPF-C, patients received cetuximab weekly combined with concurrent chemoradiation, with either weekly cisplatin 40 mg/m^2^ or weekly carboplatin AUC 1.5 mg/ml/min. The study was terminated prematurely due to mainly gastrointestinal serious adverse events [Vermorken JB, Pers. Comm.]. The DeLOS II trial is a German multicenter randomized Phase II larynx preservation study evaluating induction TPF with or without cetuximab followed by RT (Dietz et al., 2010). The primary end point of the trial was functional larynx preservation rate. Patients with no response after one cycle of induction chemotherapy were to proceed to laryngectomy. A standard TPF regimen was initially employed. However, due to five toxic deaths in the first 62 enrolled patients (4 in the TPF-alone arm), 5-Fluorouracil was subsequently omitted from the induction regimen.

Mesia et al. (2009) conducted a phase II trial in seven Spanish hospitals of four cycles of the European induction TPF regimen plus cetuximab followed by cetuximab radioimmunotherapy in patients with unresectable locally advanced-stage IV HNSCC and PS 0–1. Induction comprised docetaxel 75 mg/m^2^ on day 1, cisplatin 75 mg/m^2^ on day 1, 5-Fluorouracil 750 mg/m^2^ on days 1–5, and cetuximab 250 mg/m^2^ on days 1, 8, and 15 (initial dose 400 mg/m^2^ on cycle 1, day 1), repeated every 21 days for four cycles with prophylactic antibiotics and G-CSF support. Subsequently, patients received accelerated RT with a concomitant boost (69.9Gy) and cetuximab 250 mg/m^2^ weekly. Objective response rate to TPF-C was the primary end point. G-CSF and antibiotics were given prophylactically. Serious grade 3/4 adverse events were: neutropenia (24%); neutropenic fever (20%), infection (6%); thrombocytopenia (4%); diarrhea (12%); hepatotoxicity (4%); hypomagnesemia (2%). There were two toxic deaths due to febrile neutropenia and hepatic insufficiency. RR (CR + PR + SD) after four cycles of induction was 78%. Survival results have not been reported to date.

Here, we report our experience of treating selected fit patients with locally advanced HNSCC with induction TPF-C followed by concurrent cetuximab-radioimmunotherapy (70Gy). We used docetaxel 75 mg/m^2^, cisplatin 75 mg/m^2^, 5-Fluorouracil dose of 1000 mg/m^2^/day as a continuous infusion for 4 days and cetuximab 500 mg/m^2^ on day 1 of 21-day chemotherapy cycle. Our sequential regimen was feasible and it showed promising activity. No toxic deaths occurred. The main difference from the other TPF-C regimens is that instead of standard dose cetuximab we used 500 mg/m^2^ on day 1 of 21-day chemotherapy cycle. This modified dose and schedule of cetuximab resulted in reduced gastrointestinal toxicity. In the aforementioned DFCI study of TPF-C, despite reduction in 5-Fluorouracil dose level of standard TPF from 1000–850 mg/m^2^, an average of seven cetuximab doses were given of nine intended. The combination of 5-Fluorouracil with standard dose cetuximab resulted in excessive gastrointestinal toxicity. Cetuximab 500 mg/m^2^ on day 1 of 14-day chemotherapy cycle has been found as active as standard dose cetuximab in colon cancer. Two randomized studies were presented at American Society of Clinical Oncology Meeting in 2012 comparing concurrent chemo RT with sequential treatment regimens. Both of them were terminated before the planned accrual could be reached due to slow enrollment (Cohen et al., 2012; Haddad et al., 2012). In both studies the standard arms performed unexpectedly well probably due to HPV epidemic in the United States and oropharynx cases represented the majority of patients. The studies showed no survival difference between standard concurrent chemo RT and sequential regimens. The studies do not provide definitive answer concerning the value of induction chemotherapy. Our retrospective analysis included only two oropharynx cases which were both HPV negative.

We found no activating *PIK3CA* mutations in this small cohort of HPV negative HNSCC. Our findings are consistent with data showing that *PIK3CA* mutations are more common in HPV positive HNSCC (Yarbrough et al., 2007).

To summarize, the combination of our modified induction TPF-C regimen with concurrent cetuximab radioimmunotherapy in patients with locally advanced head and neck squamous cell carcinoma is tolerable, with encouraging efficacy.

## Conflict of Interest Statement

The authors declare that the research was conducted in the absence of any commercial or financial relationships that could be construed as a potential conflict of interest.

## Footnotes

^1^National Cancer Institute. Common Toxicity Criteria manual,version 3.0: July 16, 2003. Available from: http://ctep.cancer.gov/reporting/ctc.html. Accessed July 2006.
